# The SARS-CoV-2 Spike protein disrupts human cardiac pericytes function through CD147 receptor-mediated signalling: a potential non-infective mechanism of COVID-19 microvascular disease

**DOI:** 10.1042/CS20210735

**Published:** 2021-12-15

**Authors:** Elisa Avolio, Michele Carrabba, Rachel Milligan, Maia Kavanagh Williamson, Antonio P. Beltrami, Kapil Gupta, Karen T. Elvers, Monica Gamez, Rebecca R. Foster, Kathleen Gillespie, Fergus Hamilton, David Arnold, Imre Berger, Andrew D. Davidson, Darryl Hill, Massimo Caputo, Paolo Madeddu

**Affiliations:** 1 Bristol Medical School, Translational Health Sciences, University of Bristol, Bristol, U.K.; 2 School of Cellular and Molecular Medicine, University of Bristol, Bristol, U.K.; 3 Department of Pathology, University of Udine, Udine, Italy; 4 School of Biochemistry, University of Bristol, Bristol, U.K.; 5 Medicines Discovery Institute, Cardiff University, Cardiff, U.K.; 6 Max Planck Bristol Centre for Minimal Biology, University of Bristol, Bristol, U.K.

**Keywords:** angiotensin converting enzyme 2, CD147, COVID-19, Microvascular disease, pericyte, Spike protein

## Abstract

The severe acute respiratory syndrome coronavirus 2 (SARS-CoV-2) causes a broad range of clinical responses including prominent microvascular damage. The capacity of SARS-CoV-2 to infect vascular cells is still debated. Additionally, the SARS-CoV-2 Spike (S) protein may act as a ligand to induce non-infective cellular stress. We tested this hypothesis in pericytes (PCs), which are reportedly reduced in the heart of patients with severe coronavirus disease-2019 (COVID-19). Here we newly show that the *in vitro* exposure of primary human cardiac PCs to the SARS-CoV-2 wildtype strain or the α and δ variants caused rare infection events. Exposure to the recombinant S protein alone elicited signalling and functional alterations, including: (1) increased migration, (2) reduced ability to support endothelial cell (EC) network formation on Matrigel, (3) secretion of pro-inflammatory molecules typically involved in the *cytokine storm*, and (4) production of pro-apoptotic factors causing EC death. Next, adopting a blocking strategy against the S protein receptors angiotensin-converting enzyme 2 (ACE2) and CD147, we discovered that the S protein stimulates the phosphorylation/activation of the extracellular signal-regulated kinase 1/2 (ERK1/2) through the CD147 receptor, but not ACE2, in PCs. The neutralisation of CD147, either using a blocking antibody or mRNA silencing, reduced ERK1/2 activation, and rescued PC function in the presence of the S protein. Immunoreactive S protein was detected in the peripheral blood of infected patients. In conclusion, our findings suggest that the S protein may prompt PC dysfunction, potentially contributing to microvascular injury. This mechanism may have clinical and therapeutic implications.

## Introduction

Microvascular complications are frequent and harmful in patients with coronavirus disease-2019 (COVID-19) caused by severe acute respiratory syndrome coronavirus 2 (SARS-CoV-2), with up to 11% of those hospitalised in intensive care units having myocardial ischaemia or infarction [1–4]. Moreover, people with pre-existing cardiovascular diseases are more likely to die of COVID-19 [5]. The link between the two conditions is not completely understood, especially in the light of the controversy about the capacity of the coronavirus to infect the vascular endothelium [6–9]. While nasal and pulmonary epithelial cells are the primary target for infection, after viral replication and circulation, many other cells in distant organs, including heart resident cells, become exposed and potentially infected. Cardiac myocytes and fibroblasts express the main entry receptor angiotensin-converting enzyme 2 (ACE2), but pericytes (PCs), mural cells that support the maintenance and repair of microvasculature throughout the myocardium [10–12], appear particularly susceptible because they reportedly express the highest levels of ACE2 in the heart [13–15]. Interestingly, a reduction in the vascular coverage by PCs was documented in the heart and lungs of human patients with COVID-19, in the absence of capillary rarefaction, suggesting that SARS-CoV-2 may affect the microvasculature by specifically targeting PCs [16,17].

The biological effects of SARS-CoV-2 are governed by the interaction of the homotrimeric spike (S) glycoprotein with cognate receptors on human cells. Such binding triggers a cascade of events that leads to fusion of the viral and cellular membranes to facilitate virus entry [18], and subsequent manipulation of the host gene transcription machinery to regulate viral replication [19]. The receptor-binding domain (RBD) contained in the S1 subunit of the viral S protein recognises and binds to ACE2, while the S2 subunit mediates viral–cell membrane fusion by forming a six-helical bundle via the two-heptad repeat domain [20,21]. Interestingly, several studies have challenged the concept that ACE2 is indispensable for the S1 subunit to engage with cells and induce intracellular signalling [22–24]. CD147, also known as Basigin (*BSG*) or extracellular matrix metalloproteinase inducer (EMMPRIN), initially identified as an entry receptor for measles virus [25], has recently emerged as a novel receptor for SARS-CoV-2 [26]. Intriguingly, this transmembrane protein is expressed by endothelial cells (ECs), signals through extracellular signal-regulated kinase 1/2 (ERK1/2), is up-regulated during inflammation and atherothrombosis, and may contribute to plaque instability by inducing metalloproteinase expression [27]. All these properties suggest CD147 as a potential mediator of the cardiovascular damage caused by SARS-CoV-2.

The aims of the present study were to: (1) explore if human cardiac PCs express ACE2 or CD147, or both; (2) verify if PCs are permissive to SARS-CoV-2 infection *in vitro*; (3) investigate whether a recombinant SARS-CoV-2 S protein alone, outside the context of infectious virus, can trigger molecular, functional, and pro-inflammatory alterations in PCs; (4) to adopt blocking antibodies or mRNA silencing strategies to understand which viral receptor is responsible for the harmful S protein effects in PCs. Moreover, we verified the presence of immunoreactive S protein in the blood samples of COVID-19 patients.

## Methods

Unless otherwise stated, all chemicals were purchased from Sigma–Aldrich, U.K.

### Ethics

The present study complies with the ethical guidelines of the Declaration of Helsinki.

#### Histology on human hearts

Myocardial samples collection was covered by the Independent Ethics Committee of the University Hospital of Udine (22 October 2013, ref. 58635). Patients (*n*=3) were enlisted for cardiac transplant or device implantation due to end-stage heart failure. All patients signed informed consent. Patients were recruited before the COVID-19 pandemic.

#### Extraction of primary cardiac PCs

Human myocardial samples were discarded material from surgical repair of congenital heart defects (ethical approval number: 15/LO/1064 from the North Somerset and South Bristol Research Ethics Committee). Adult patients and paediatric patients’ custodians gave informed written consent. Donors and samples characteristics are described in Table 1. Patients were recruited before the COVID-19 pandemic.

**Table 1:** Cardiac PCs donors

Cell source	Congenital heart defect/intervention needed	Patient age
RV	Pulmonary atresia	11 days
RA	Total anomalous pulmonary vein connection	18 days
RV	Ventricular septal defect	6 months
RV	Tetralogy of Fallot	6 months
RV	Atrial–ventricular canal closure	3 years
LV	Obstructive hypertrophic cardiomyopathy – mitral valve repair	6 years
RV	Pulmonary valve repair	14 years
RV	Tetralogy of Fallot	14 years
RV	Pulmonary valve repair	15 years
RA	Atrial septal defect	17 years
RV	Tricuspid valve replacement + Pulmonary valve repair	23 years
RA	Atrial septal defect	54 years

Abbreviations: LV, left ventricle; RA, right atrium; RV, right ventricle.

#### Serological studies

Serum samples from COVID-19 patients (*n*=64; 32 M/32 F; age range: 20–93 years) were collected as part of the DISCOVER study from patients admitted to North Bristol NHS Trust (Ethics approval via South Yorks REC: 20/YH/0121, CRN approval number: 45469). Blood was withdrawn between 0 and 34 days from the onset of COVID-19 symptoms. Pre-pandemic control sera (*n*=14; 3 M/11 F; age range: 30–69 years) were randomly selected from 526 anonymised blood donor samples (age range: 18–69 years; 277 M/249 F). Ethical approval number 19/WA/0295 was granted by Wales 6 REC. All donors and patients gave informed written consent.

### Immunofluorescence analysis of cardiac PCs *in situ*

Human myocardial samples were either fixed in formalin and paraffin-embedded, or frozen using OCT compound. Five-micrometre-thick sections were cut for identification of cardiac PCs *in situ*. Paraffin sections required heat-induced antigen retrieval, performed using citrate buffer 0.01 M pH = 6, for 40 min at 98°C. Tissue sections were blocked with 10% v/v normal donkey serum and incubated with primary antibodies for 16 h at 4°C. Antibodies were: anti-CD34 (Dako M7165, 1:100), anti-CD31 (Abcam ab28364, 1:50), anti-platelet derived growth factor receptor β (PDGFRβ – R&D AF385, 1:50), anti-smooth muscle α-actin (α-SMA – Dako GA611, 1:100), anti-ACE2 (Merck SAB3500346, 1:40), anti-CD147 (BioLegend 306221, 1:100), anti-von Willebrand Factor (vWF – Merck F3520, 1:200). Donkey secondary antibodies (Alexa 488-, Alexa 568-, Alexa 647-conjugated) were all purchased from Thermo Fisher Scientific and used at a dilution of 1:200, for 1 h at 20°C in the dark. Slides were mounted using ProLong™ Gold Antifade Mountant with 4′,6-diamidino-2-phenylindole (DAPI) (Thermo Fisher Scientific). Imaging was performed using a Leica TCS SP8 confocal microscope and a Leica SP5-II AOBS multi-laser confocal laser scanning microscope attached to a Leica DM I6000 inverted epifluorescence microscope (Leica Microsystems).

### Primary cell cultures

Cardiac PCs were immunosorted as CD31neg/CD34pos cells from human myocardial samples, and expanded in a dedicated medium supplemented with human recombinant growth factors and 2% v/v foetal calf serum (FCS) (ECGM2 complete kit, C-22111, PromoCell) as previously described [11,28]. Briefly, samples were finely minced using scissors and scalpel until nearly homogenous and digested with Liberase (Roche) for up to 1 h at 37 C, with gentle rotation. The digest was passed through 70-, 40-, and 30-μm strainers. Finally, the cells were recovered and sorted using anti-CD31 and -CD34 microbeads (Miltenyi) to deplete the population of CD31pos ECs and select CD31neg/CD34pos cells, which distinguish a population of perivascular cells *in situ* [11,28]. After expansion to passage 3, the purity of the cell population was verified using immunocytochemistry (ICC) or flow cytometry [11,28].

Human coronary artery ECs (CAECs) were purchased from PromoCell and expanded in the same medium used for PCs. All cells used in the present study tested negative for mycoplasma contamination (assessed using the PCR Mycoplasma Test Kit I/C, PromoCell, cat# PK-CA91-1096). Cells were used between passages 4 and 7.

### Cell line cultures

The human gut epithelial cell line, Caco2, expressing hACE2 (Caco-2-ACE2) was a kind gift from Dr Yohei Yamauchi, University of Bristol. The African green monkey kidney cell line VeroE6 engineered to overexpress the human ACE2 and TMPRSS2 (VeroE6/ACE2/TMPRSS2) [29] was a kind gift from Dr Suzannah Rihn, MRC-University of Glasgow Centre for Virus Research. All cells were cultured in Dulbecco’s modified Eagle’s medium plus GlutaMAX (DMEM, Gibco, Thermo Fisher, cat# 10567014) supplemented with 10% v/v FBS (Gibco, Thermo Fisher, A3840001), 1% v/v sodium pyruvate, and 0.1 mM non-essential amino acids. The human lung epithelial cell line Calu3 (ATCC HTB-55) was cultured in Eagle’s minimum essential medium plus GlutaMAX (MEM, Gibco, Thermo Fisher, cat# 41090036) with 10% v/v FBS, 0.1 mM non-essential amino acids, and 1% v/v sodium pyruvate.

### ICC analyses

Cells were rinsed with phosphate-buffered saline (PBS) and fixed with 4% w/v paraformaldehyde (PFA) in PBS for 15 min at 20°C. After washing with PBS, the cells were permeabilised with 0.1% v/v Triton-X100 in PBS for 5 min at 20°C, when required. Cells were blocked with 10% v/v normal donkey serum (Abcam ab7475) and incubated with the following antibodies for 16 h at 4°C: anti-ACE2 (R&D AF933, dilution 1:50); anti-CD147 (BioLegend 306221, 1:100); anti-Transmembrane Serine Protease 2 (TMPRSS2 – Proteintech 14437-1-AP, 1:100); anti-Neural/Glial antigen 2 (NG2 – Millipore AB5320, 1:100); anti-PDGFRβ (R&D AF385, 1:100); anti-PDGFRα (Santa Cruz sc-398206, 1:100); anti-CD34 (Dako M7165, 1:100); anti-CD31 (Abcam ab28364, 1:50). Donkey secondary antibodies conjugated with either Alexa 488 or Alexa 568 or Alexa 647 were purchased from Thermo Fisher Scientific and used at a dilution of 1:200, for 1 h at 20°C, in the dark. Nuclei were counterstained using DAPI. Images were snapped and processed using a Zeiss AxioObserver Z1 Microscope equipped with a 20× objective.

### Western blotting on total cell lysates

Whole-cell protein lysates were prepared using RIPA buffer supplemented with 1:50 proteases’ inhibitors cocktail and 1:100 phosphatases’ inhibitors. Protein extracts were centrifuged 15 min at 10000×***g***, 4°C. After the assessment of protein concentration (BCA Protein Assay Kit, Thermo Fisher Scientific), the supernatants were kept at −80°C. Protein samples (10–15 μg) were prepared in Laemmli loading buffer, incubated for 8 min at 98°C, resolved on 10% SDS/PAGE, and transferred on to 0.2-μm PVDF membrane (Bio-Rad). Membranes were blocked using 5% w/v non-fat dried milk (Bio-Rad) in Tris-buffered saline (TBS, Bio-Rad) supplemented with 0.05% v/v Tween-20 for 2 h at 20°C. Primary antibodies (ACE2, dilution 1:100; TMPRSS2, 1:1000; CD147, 1:500; 6×-HIS-tag (Invitrogen MA1-21315), 1:1000; P-ERK1/2 Thr^202^/Tyr^204^ (Cell Signaling Technology #4370), 1:2000; Total ERK1/2 (Cell Signaling Technology #4395), 1:1000) were incubated for 16 h at 4°C. GAPDH was used as a loading control (Cell Signaling Technology #97166, 1:1000). Anti-mouse or anti-rabbit IgG HRP (1:5000, both from GE Healthcare) or anti-goat IgG HRP (R&D HAF017, 1:5000) were employed as secondary antibodies. Membrane development was performed by an enhanced chemiluminescence-based detection method (ECL™ Prime Western Blotting Detection Reagent, GE Healthcare) and observed using a ChemiDoc MP Imaging System (Bio-Rad). Western blot data were analysed using the Bio-Rad Image Lab and the ImageJ software.

For detection of the S protein binding to PCs, 1 μg/ml (5.8 nM) S protein or PBS vehicle were incubated with PCs for 1 h at 37°C, and whole cell protein lysates collected in RIPA buffer as described.

### Infections of primary cardiac PCs and Caco-2 cells with SARS-CoV-2

Stocks of SARS-CoV-2 viral isolates, SARS-CoV-2/human/Liverpool/REMRQ0001/2020 (REMRQ0001, wildtype strain, GenBank: MW041156.1, isolated as previously described [30]), hCoV-19/England/204690005/2020 (lineage B.1.1.7 – α variant; GISAID ID: EPI_ISL_693401, kindly provided by Professor Wendy Barclay, Imperial College, London and Professor Maria Zambon, Public Health England), and hCoV-19/England/SHEF-10E8F3B/2021 (lineage B.1.617.2 – δ variant; GISAID ID: EPI_ISL_1731019; kindly provided by Professor Wendy Barclay, Imperial College, London and Dr Thushan de Silva, Sheffield Teaching Hospitals, University of Sheffield) were produced by inoculation of VeroE6/TMPRSS2 cells [31] and titred as previously described [32,33]. Caco-2-ACE2 cells and cardiac PCs were plated in µClear 96-well microplates (Greiner Bio-one) and the next day infected with either REMRQ0001, B.1.1.7 or B.1.617.2 at a multiplicity of infection (MOI) of 10, using respective culture media, and incubated at 37°C. Uninfected controls which received media only were also included. *n*=6 patients’ PCs were tested. Per each patient, experiments were performed in technical triplicates, except the δ virus infections which were performed in duplicate.

After 24 h, cells were fixed with 4% w/v PFA for 60 min before being permeabilised with 0.1% v/v Triton-X100 and blocked with 1% v/v bovine serum albumin (BSA). Cells were stained with DAPI and antibodies against double-stranded RNA (dsRNA, J2 10010200, Scicons) and SARS-CoV-2 nucleocapsid (N) protein (200-401-A50, Rockland) followed by appropriate secondary antibodies conjugated with Alexa Fluor dyes 586 and 647. For staining with goat anti-human PDGFRβ antibody (same as above), after fixing, cells were blocked in 5% donkey serum in PBS, then antibody was added at a 1:50 dilution and incubated overnight at 4°C. A secondary antibody conjugated with Alexa Fluor dye 488 was used. For quantification, an ImageXpress Pico Automated Cell Imaging System (Molecular Devices) was used to capture immunofluorescence using a 10× objective. Cells were determined to be uninfected if less than 1% of the cells were dsRNA antibody-positive. Representative images were captured using a Zeiss AxioObserver Z1 Microscope equipped with a 20× objective. All work with infectious SARS-CoV-2 virus was conducted in a Class III microbiological safety cabinet in a containment level 3 facility at the University of Bristol.

### Measurement of S protein in patients’ sera

The presence of S protein in COVID-19 patients’ serum was evaluated using the COVID-19 Spike Protein ELISA Kit from Abcam (ab274342), according to manufacturer’s instructions. Pre-pandemic sera were employed as controls. All test sera were diluted 1:2. The S protein concentration was expressed as nanogram per millilitre serum. The antibody supplied in the kit recognised the S2 domain.

### Abbott SARS-CoV-2 IgG assay

The presence of anti-SARS-CoV-2 N protein IgG antibodies in patients’ serum was evaluated using the Abbott SARS-CoV-2 IgG assay (Abbott Laboratories) [34]. This kit is a chemiluminescent microparticle assay used for the qualitative detection of IgG antibodies to SARS-CoV-2 N on the Architect system. The outcome is a calculated Index reflecting the amount of IgG antibodies in the sample. The index cutoff is 1.4, with samples equal or above this value considered positive for anti-SARS-CoV-2 N antibodies, and samples below this threshold considered negative.

### Production and purification of the recombinant SARS-CoV-2 S protein

SARS-CoV-2 S protein was expressed in insect cells and purified as described previously [33,35]. Briefly, the S construct encoded amino acids 1–1213 (extracellular domain – ECD) fused with a thrombin cleavage site, followed by a T4-foldon trimerisation domain and a hexahistidine (HIS) affinity purification tag at the C-terminus. The polybasic furin cleavage site was mutated (RRAR to A) to increase the stability of the protein for *in vitro* studies [33,35]. S protein was expressed in Hi5 cells using the MultiBac system [36]. Secreted S protein was harvested 3 days after infection by centrifuging the cell culture at 1000*×**g*** for 10 min followed by another centrifugation of supernatant at 5000*×**g*** for 30 min. S protein-containing medium was incubated with HisPur Ni-NTA Superflow Agarose (Thermo Fisher Scientific) for 1 h at 4°C. Resin bound with S protein was separated from unbound proteins and medium using a gravity flow column, followed by 30 column volume wash with wash buffer (65 mM NaH_2_PO_4_, 300 mM NaCl, 20 mM imidazole, pH 7.5). Finally, the protein was eluted with a step-gradient of elution buffer (65 mM NaH_2_PO_4_, 300 mM NaCl, 235 mM imidazole, pH 7.5). Eluted fractions were analysed by reducing SDS/PAGE. Fractions containing the S protein were pooled and concentrated using 50-kDa MWCO Amicon centrifugal filter units (EMD Millipore). During concentration, proteins were buffer-exchanged in PBS, pH 7.5. Concentrated protein was aliquoted, flash-frozen in liquid nitrogen, and stored at −80°C until use. In all the *in vitro* experiments of the manuscript, we will refer to the S-ECD protein simply as S protein.

Recombinant Spike S1 (#10522-CV) and S2 (#10584-CV) were purchased from R&D, resuspended in PBS according to manufacturer’s instructions, aliquoted and stored at −80°C until use. Similarly to the S-ECD, the S1 and S2 proteins were produced in insect cells.

### Wound closure migration assay

Cells were seeded into 96-well plates. A scratch was produced in confluent PCs in the centre of each well using a 20-μl tip. Cells were washed twice with PBS to remove detached cells and incubated with EBM2 medium under FBS and growth factors deprivation during the experiment. Cell proliferation was inhibited using hydroxyurea (2 mM). Where required, cells were pre-incubated with the anti-CD147 antibody (20 μg/ml) for 1 h at 37°C. In selected experiments, a mouse IgG1 k (Thermo Fisher, cat# 12-4714-42) was used as an isotype control at the same concentration of the CD147 antibody. For the initial dose–response experiment, increasing concentrations of S protein were employed (62.5, 125, 250, 500, 1000 ng/ml). For all the following experiments, the S proteins (either S or S1 or S2) were added to the system at a final concentration of 5.8 nM. PBS vehicle was used as control. Images were snapped at baseline and after 24 h, using an inverted Leica microscope equipped with a 5× objective. The wound area was measured, and the percentage of wound closure calculated. Per each patient, experiments were performed in four to five replicates.

### Assessment of cell viability

Viability of cardiac PCs and CAECs exposed to 1 μg/ml (5.8 nM) S protein or PBS vehicle was evaluated using the Viability/Cytotoxicity Assay Kit (Biotium #30002), according to manufacturer’s guidelines. Cytoplasmic Calcein-AM identified live cells, while nuclear Ethidium Homodimer III (EthD-III) the dead cells. Cells treated with 0.1% w/v saponin for 10 min served as positive control for EthD-III staining. Per each patient, experiments were performed in duplicates.

### Assessment of cell proliferation

The Click-iT EdU Cell Proliferation Kit for imaging (C10337 – ThermoF isher Scientific) was used to assess cell proliferation, according to the manufacturer’s instructions. Cells were incubated with EdU for 24 h in the presence of S protein (1 μg/ml – 5.8 nM) or PBS vehicle, and then analysed. Per each patient, experiments were performed in duplicates.

### 2D-Matrigel angiogenesis assay

Human CAECs were seeded on the top of Matrigel (Corning® Matrigel® Growth Factor Reduced Basement Membrane Matrix, cat# 356231) either in monoculture (4000 cells/well) or in coculture with PCs (4000 CAECs + 1500 PC/well), using Angiogenesis μ-Slides (IBIDI, U.K.) and growth factors-free medium. The S protein (1 μg/ml – 5.8 nM) or PBS vehicle were added to the system. Images were taken after 5 h using an inverted Leica microscope equipped with a 5× objective. The total tube length per imaging field was measured using ImageJ. To assess the interaction between PCs and CAECs, PCs were labelled with the red fluorescent tracker *Vybrant*™ *DiI Cell-Labeling Solution* (Invitrogen; dilution 1:1000 in PBS, incubation for 5 min at 37°C followed by 15 min at 4°C). For experiments requiring the CD147 blockade, 100000 PCs in a total volume of 100 μl were pre-incubated with the anti-CD147 antibody (20 μg/ml) for 1 h at 20°C. Per each patient, experiments were performed in triplicates.

### Assessment of ERK1/2 phosphorylation

For detection of ERK1/2 phosphorylation in cardiac PCs, either Western blotting or the ERK1/2 ELISA® Kit (Abcam ab176660) were employed. PCs were cultured with EBM2 medium under FBS and growth factors deprivation for 24 h. When required, PCs were pre-incubated with the anti-ACE2 (20 μg/ml, as described before [20]) or anti CD147 (20 μg/ml) antibodies for 1 h at 37°C. Cells were exposed to the Spike protein (5.8 nM) or PBS vehicle for 1 h at 37°C. Lysates were collected for western blotting or ELISA. Per each patient, experiments were performed in duplicates.

### CD147/*BSG* silencing in cardiac PCs

Opti-MEM media (cat# 11058-021, Thermo Fisher Scientific, U.K.) and Lipofectamine RNAiMAX Transfection Reagent (cat# 13778030, Invitrogen, U.K.) were used to transfect PCs with On-target plus *BSG* SMARTpool (a mixture of four siRNAs in a single reagent, L-010737-00-0005, Dharmacon, Horizon Discovery, U.K.). The siGENOME Non-Targeting siRNA Pool #1 (Dharmacon, Horizon Discovery, U.K.) was used as a control (final concentration 25 nM for both). The transfection reagent was removed after 6 h and replaced with fresh EGM2 medium. On days 3 and 4 post-transfection, cells were used for functional assays. RNA and total cell lysates were collected, and silencing was confirmed using qPCR, ICC, and Western blotting.

### Gene expression analysis by real-time qPCR

Extracted total RNA was reverse-transcribed into single-stranded cDNA using a High-Capacity RNA-to-cDNA Kit (Applied Biosystems, cat# 4387406, Thermo Fisher Scientific). RT-PCR was performed using first-strand cDNA with TaqMan Fast Universal PCR Master Mix (Applied Biosystems, cat# 4352042, Thermo Fisher Scientific). TaqMan primer-probes were obtained from Thermo Fisher Scientific (*BSG* Hs00936295_m1; housekeeping gene *UBC* Hs00824723_m1). Quantitative PCR was performed on a QuantStudio™ 5 System (Thermo Fisher). All reactions were performed in a 10 μl volume in triplicate, using 7.5 ng cDNA per reaction. The mRNA expression levels were normalised against UBC and determined using the 2^−ΔΔ*C*_t_^ method [37].

### Collection of PC conditioned medium

For medium collection, confluent PCs were maintained for 24 h in serum- and growth factors-free medium. When required, cells were pre-incubated with the anti-CD147 antibody for 1 h (20 μg/ml). The S protein (1 μg/ml – 5.8 nM) or PBS vehicle were added for 24 h. Conditioned media were collected and centrifuged for 10 min at 1000×***g***, at 4°C, and stored at −80°C until use.

### Cytokines and chemokines array

A panel of 105 citokines/chemokines was assessed in the conditioned medium of cardiac PCs treated with either the Spike protein or PBS vehicle, using the Human XL Cytokine Array kit (R&D, ARY022B). Each membrane was incubated with 750 μl conditioned medium. The assay was carried out according to manufacturer’s instructions. Blot densitometry data for Spike-treated PCs are reported as fold-change versus the respective vehicle.

### Measurement of cytokines using ELISA

Monocyte chemoattractant protein-1 (MCP1/CCL2, R&D DY279), tumour necrosis factor (TNFα, R&D DY210), interleukin (IL)-6 (IL-6, R&D DY206), IL-1β (R&D DLB50) were measured in PC conditioned medium using ELISA. Per each patient, experiments were performed in triplicates. Cell protein extracts were collected using RIPA buffer and the total amount of protein was quantified for normalisation of secreted factors (BCA assay). Secreted factors were expressed as pg secreted protein per 100 μg of total cellular protein.

### Effect of PC secretome on EC apoptosis

CAECs were seeded in 96-well plates. After 24 h, cells were incubated for additional 24 h with the PC conditioned medium diluted 1:2 with fresh EBM2 growth factors-free medium, with a final serum concentration of 1%. CAECs were also incubated with the S protein (1 μg/ml – 5.8 nM) or PBS vehicle for 24 h. Cell apoptosis was assessed using either the CaspaseGlo 3/7 assay (G8090, Promega, U.K.) according to the manufacturer’s instructions (*n*=4 replicates/patient), or the Calcein-AM/EthD-III viability kit (Biotium) (in duplicate per each patient). EC death was measured either as caspase activity (relative luminescence units) or as % of EthD-III-positive cells, and finally expressed as fold changes versus the control vehicle group.

### Statistical analyses

Data were analysed using Prism version 8.0 and expressed as individual values and as means ± standard error of the mean (SEM). For cell biology experiments, because the sample size was not big enough to apply the normality tests, non-parametric tests were used. For analysis of patients’ clinical data, the D’Agostino–Pearson and Kolmogorov–Smirnov normality tests were used to check for normal distribution. Statistical differences were determined using unpaired *t* tests, or one- or two-way ANOVAs, as appropriate. Statistical significance was assumed when *P*≤0.05.

## Results

### Cardiac PCs express SARS-CoV-2 S protein receptors

**Figure 1:**
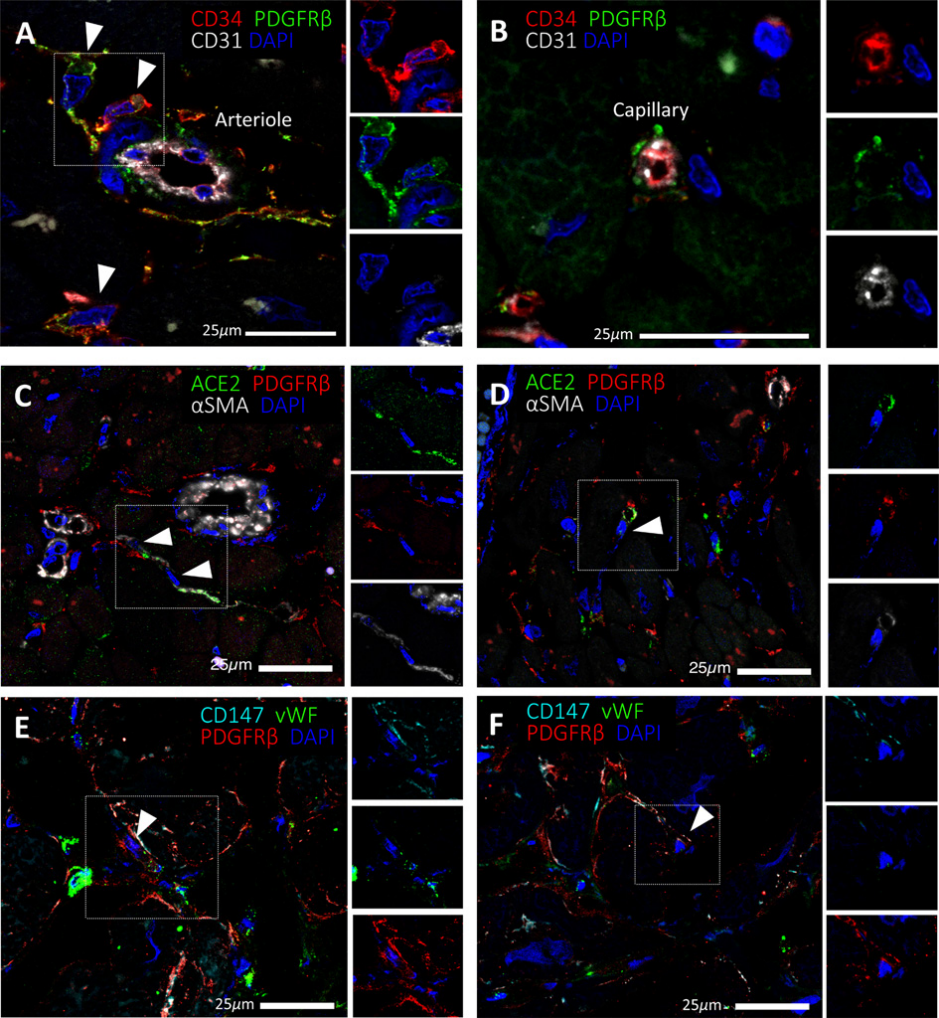
Expression of SARS-CoV-2 receptors in human cardiac PCs *in situ* Immunofluorescence stainings showing PCs in the human heart. (**A**,**B**) Identification of CD31^−^CD34^+^PDGFRβ^+^ PCs around microvessels. CD31 recognises the vessel lumen, the CD34 labels both the luminal ECs and perivascular PCs, while PDGFRβ labels both PCs and vascular smooth muscle cells (VSMCs) in the arteriole’s tunica media. (**C–F**) A subset of PDGFRβ^+^ cardiac PCs express ACE2 (C,D) and CD147 (E,F). α-SMA labels some PCs and arterioles’ VSMCs (C,D), while vWF recognises ECs (E,F). CD147 is also expressed by ECs (E,F). Arrowheads point to PCs.

We previously described a population of CD31^−^ CD34^+^ PDGFRβ^+^ vascular PCs around microvessels of the human heart (Figure 1A,B) [11]. Here we have further confirmed that a subset of PDGFRβ^+^ PCs express the S protein receptor ACE2 *in situ* (Figure 1C,D). We also show for the first time that human cardiac PCs express the alternative receptor CD147 (Figure 1E,F).

**Figure 2:**
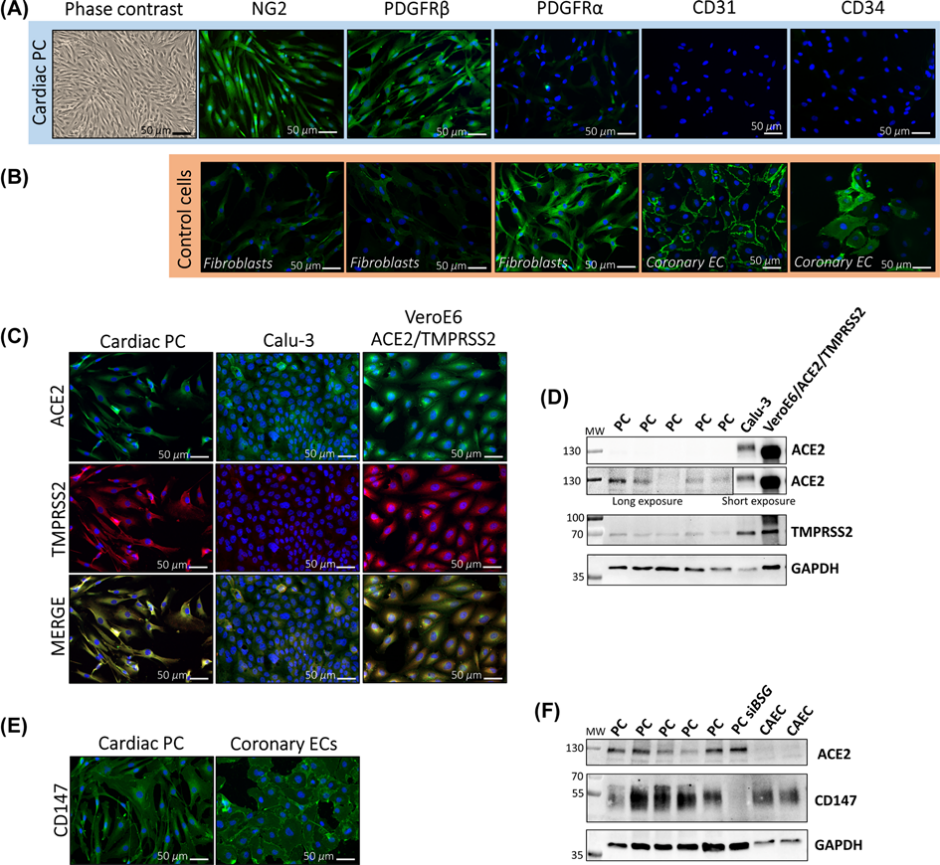
Expression of the SARS-CoV-2 receptors ACE2 and CD147, and activating protease TMPRSS2, in cultured cardiac PCs (**A**) Contrast-phase and immunofluorescence images showing the characteristic shape and antigenic phenotype of cultured cardiac PCs. In green fluorescence, the antigens as indicated. In blue nuclei (DAPI), NG2: Neural/glial antigen 2. PDGFRα: platelet derived growth factor receptor α. (**B**) Immunofluorescence images of human cardiac fibroblasts and CAECs employed as negative or positive controls for markers in (A). (**C**) Expression of ACE2 (green) and TMPRSS2 (red) in cardiac PCs and control cells assessed by immunostaining. In blue nuclei (DAPI). Calu-3: human lung epithelial cell line. *VeroE6/ACE2/TMPRSS2*: African green monkey kidney cell line engineered to overexpress the human ACE2 and TMPRSS2. (**D**) Expression of ACE2 and TMPRSS2 assessed using Western blotting. *n*=5 patients’ PCs, *n*=1 for cell lines. (**E**) Expression of CD147 (green) in cardiac PCs and control human CAECs assessed by immunostaining. In blue nuclei (DAPI). (**F**) Expression of CD147 and ACE2 determined using Western blotting. *n*=5 patients’ PCs. *n*=2 CAEC. si*BSG* = PC in which *BSG* (CD147) was silenced, used as a negative control for CD147.

We then verified the expression of S receptors in primary cultures of cardiac PCs *in vitro*. PCs were immunosorted as CD31^−^ CD34^+^ cells from myocardial leftovers of patients undergoing heart surgery [11]. After expansion, PCs showed the characteristic spindle-shape and expressed the typical mural cell antigens NG2 and PDGFRβ, while being negative for the fibroblast marker PDGFRα, the endothelial marker CD31, and CD34 (Figure 2A). This latter, cell surface antigen expressed by PCs *in situ*, was expectedly down-regulated upon culture *in vitro*, as we previously documented for vascular PCs [11,38]. Cardiac fibroblasts and CAECs were employed either as negative or positive controls for the PC immunostaining (Figure 2B). ICC showed that PCs express the major SARS-CoV-2 receptor ACE2 as well as TMPRSS2, a coreceptor required for proteolytic activation of the S protein [20] (Figure 2C). Calu-3 and VeroE6/ACE2/TMPRSS2 cells were used as positive controls. Western blotting further indicated that cardiac PCs express considerably lower levels of both ACE2 and TMPRSS2 than control cells (Figure 2D). PCs also express CD147 (Figure 2E,F). For the last antigen, primary human CAECs were used as positive control.

### Cardiac PCs display a very low permissiveness to SARS-CoV-2 infection *in vitro*

**Figure 3:**
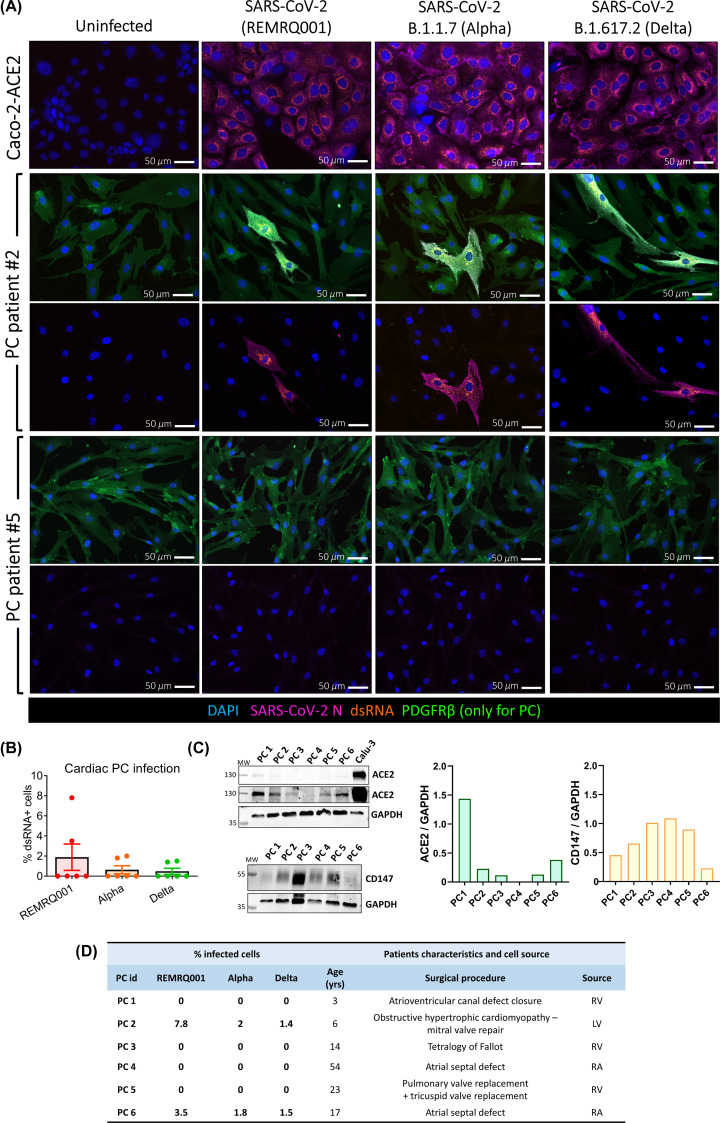
Infection of cardiac PCs by SARS-CoV-2 is a rare event *in vitro* Primary cardiac PCs (*n*=6 patients) and the Caco-2-ACE2 cell line were either mock-infected or inoculated with SARS-CoV-2 isolated early in the pandemic (REMRQ001) or the α (B.1.1.7) or δ (B.1.617.2) variants, all at an MOI = 10, and incubated for 24 h before immunostaining for viral and PC markers. (**A**) Immunofluorescence images show SARS-CoV-2 nucleocapsid protein (N, magenta) and double-stranded RNA (dsRNA, orange) indicative of virus replication. Nuclei are stained with DAPI (blue). PDGFRβ stains PCs (green). For PCs, we show example images from two patients. Because of the high magnification used (20×), the images shown for patient #2 are not representative of the real % of infection, but we aimed to provide examples of infected PCs in each experimental group. (**B**) Quantification of the percentage of PCs positive for dsRNA in the three experimental groups. The bar graph reports individual values and means ± SEM. (**C**) Analysis of ACE2 and CD147 protein levels in cardiac PCs using Western blotting. (**D**) Table showing the row data of the PC infection and summarising the patients’ characteristics and cell source. Abbreviations: LV, left ventricle; RA, right atrium; RV, right ventricle.

Next, we investigated whether SARS-CoV-2 infects cardiac PCs *in vitro*. We employed SARS-CoV-2 isolated early during the pandemic (REMRQ0001) as well as the α (B.1.1.7) and δ (B.1.617.2) variants (MOI = 10 for all). Permissive Caco-2-ACE2 cells were used as a positive control. Twenty-four hours post-inoculation, immunostaining for the N protein and dsRNA documented replicative infection only in PCs from two out of six patients (Figure 3A), with 1.4–7.8% cells showing positivity for dsRNA (Figure 3B). The quantification of SARS-CoV-2 receptors, measured using Western blot, did not show a correlation between the protein levels of ACE2 and CD147 and the cell susceptibility to infection (Figure 3C). The characteristics of patients and myocardial sources for the PCs used in this experiment are supplied in Figure 3D.

### Presence of SARS-CoV-2 S protein in the peripheral blood of COVID-19 patients

**Figure 4:**
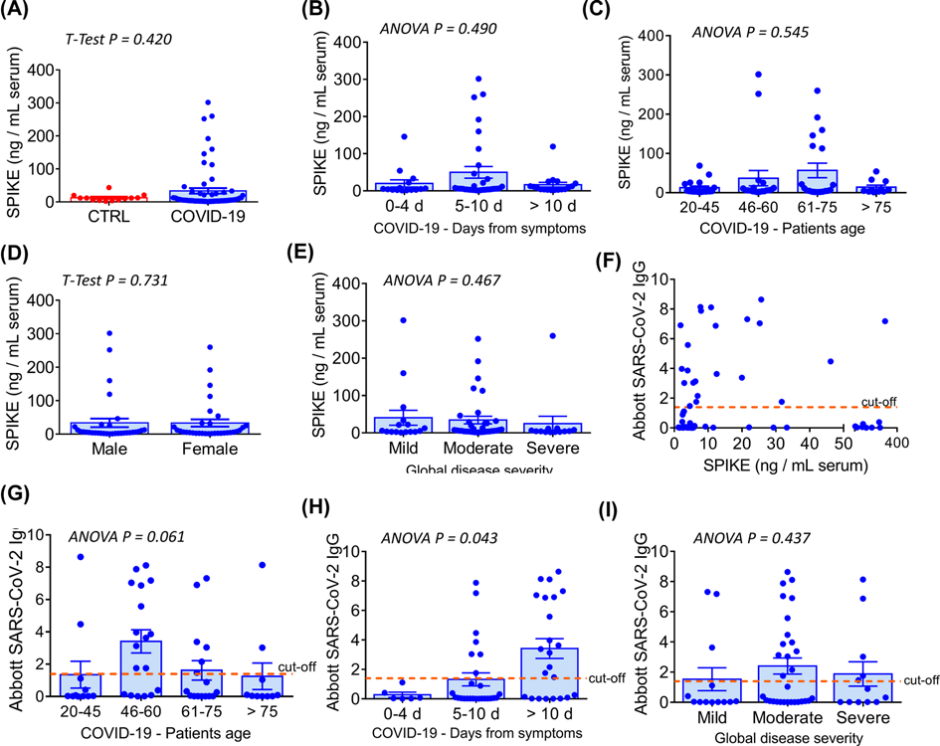
Serological analyses in COVID-19 patients and correlation with clinical data (**A**) Serological S protein concentration in pre-pandemic controls (*n*=14) vs COVID-19 patients (*n*=64). (**B**) Distribution of S protein concentration in patients according to the time from the onset of disease symptoms. (**C**) Distribution of S protein concentration according to patients’ age. (**D**) Distribution of S protein concentration according to patients’ gender. (**E**) Distribution of S protein concentration according to the global COVID-19 severity. This latter was categorised based on the patients’ respiratory symptoms as follows: *mild*: no oxygen required; *moderate*: oxygen required; *severe*: admission to intensive care or high-dependency unit (non-invasive ventilation). (**F**) Analysis of correlation between the S protein concentration and the Abbott anti-SARS-CoV-2 IgG index. The latter indicates a qualitative measurement of anti-nucleocapside (N) protein IgG antibodies. (**G**) Distribution of the Abbott anti-SARS-CoV-2 IgG index according to patients’ age. (**H**) Distribution of the Abbott anti-SARS-CoV-2 IgG index according to the time from the onset of disease symptoms. (**I**) Distribution of the Abbott anti-SARS-CoV-2 IgG index according to the global COVID-19 severity. Graphs in (A–E) and (G–I) report individual values and means ± SEM.

Using a highly sensitive ELISA, we detected the presence of immunoreactive S protein in the serum of COVID-19 patients (mean ± SEM: 33.5 ± 8.3 ng/ml), with 18 out of 64 cases having values above the 95^th^ percentile of the control population (>20.9 ng/ml), which comprised samples collected before the pandemic (Figure 4A). Elevated S protein levels prevailed in the patients’ group that was sampled between 5 and 10 days from the onset of COVID-19 symptoms (Figure 4B) and in those aged 46 or more (Figure 4C). Across groups classified according to age and symptoms, the concentration of S protein was similar between male and female (Figure 4D). Moreover, there was no correlation between the S protein concentration and disease severity, which was classified according to respiratory symptoms (Figure 4E – see legend), or the Abbott anti-SARS-CoV-2 N IgG index, which is used to assess serological response and viral neutralisation (Figure 4F). The presence of anti-N IgG was documented mainly in patients aged 46 or more (Figure 4G), and as expected, the immunological index tended to be higher at later times after the onset of the COVID-19 symptoms (Figure 4H). Finally, there was no correlation between the Abbott IgG index and the severity of the disease (Figure 4I).

### The SARS-CoV-2 S protein interacts with and causes dysfunction of cardiac PC

**Figure 5:**
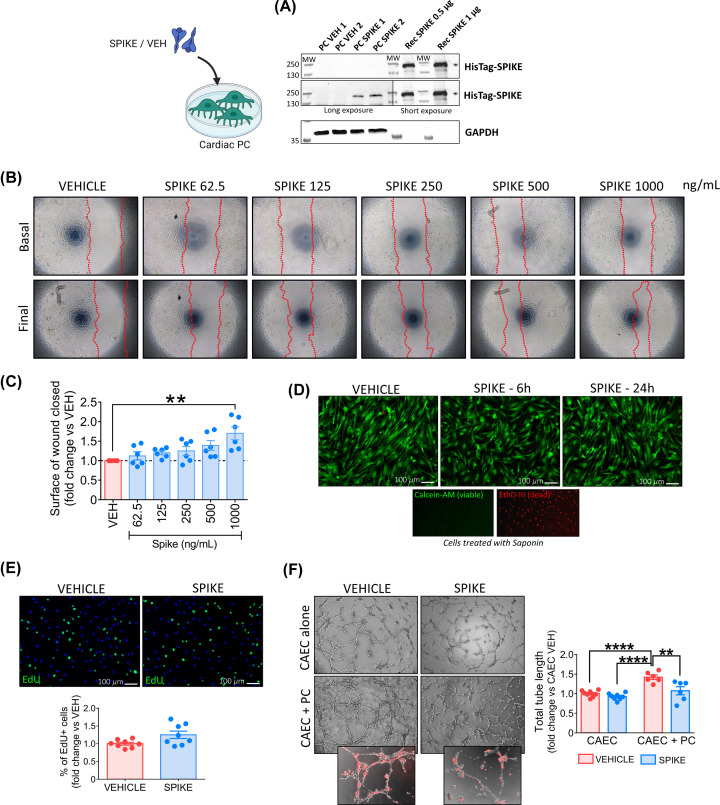
The SARS-CoV-2 recombinant S protein interacts with cardiac PCs and induces functional alterations (**A**) S protein interaction with PC receptors. Western blotting analysis of PCs (*n*=2 patients) exposed to the S protein (SPIKE) or PBS vehicle (VEH) for 1 h. The bands corresponding to the 6×-His-Tag recognise the His-tagged S protein. The purified S protein was used as a positive control. (**B,C**) Dose–response migration wound closure assay. A scratch was created in confluent PCs and images taken at baseline. Cells were incubated with increasing concentrations of S protein or vehicle for 24 h and final images were recorded. The surface of wound closure was calculated as % of the baseline area and expressed as fold-change vs vehicle. *n*=6 patients’ PCs. (**D**) Cell viability. Live cell imaging of PCs after 6 and 24 h incubation with the S protein (1 μg/ml – 5.8 nM). In green, Calcein-AM shows the cytoplasm of live cells. The red fluorescence of EthD-III indicates the nuclei of dead cells (not detected). Saponin treatment, used as a positive control for dead cells, shows the nuclear staining of EthD-III in the absence of Calcein-AM. Images are representative of one patient. The assay was done in *n*=3 patients’ PCs. (**E**) Cell proliferation. PCs were exposed to the S protein (1 μg/ml – 5.8 nM) or vehicle for 24 h, in the presence of EdU. Proliferation was measured as the % of EdU+ cells and data expressed as fold-change vs vehicle. Immunostaining shows EdU+ cells in green, nuclei (DAPI) in blue. *n*=8 patients’ PCs. (**F**) Matrigel assay. CAECs and cocultures of CAEC + PCs were incubated on the top of Matrigel for 5 h, in the presence of the S protein (1 μg/ml – 5.8 nM) or vehicle. PCs were labelled with the red fluorescent tracker CM-Dil to assess the interaction with ECs (small inserts). Graphs report the total tube length per imaging field, expressed as fold-change vs CAEC vehicle. *n*=6 patients’ PCs. All graphs report individual values and means ± SEM. **P<0.01, ****P<0.0001.

We next verified whether a recombinant ECD of the S protein binds to cardiac PCs. The recombinant S protein was tagged with an HIS sequence for easy detection. Using Western blotting, we found bands corresponding to the HIS-tagged S protein in PCs exposed to the protein for an hour (Figure 5A). The recombinant S protein was used as a positive control.

Next, we asked if this binding could trigger intracellular molecular events and functional phenomena. Under normal conditions, PCs act as stationary cells that interact with ECs to preserve vascular stability. PC detachment and migration from underlying vessels occurs in response to stress and may be responsible for the reduced PC coverage reported in the heart and lung microvasculature of COVID-19 patients [16,17]. Therefore, we interrogated the S protein capacity to trigger PC motility. Using a wound closure assay with increasing amounts of S protein, we showed a dosage of 1000 ng/ml (corresponding to 5.8 nM) induced an increase in PC migration compared with vehicle (*P*<0.01) (Figure 5B,C). Exposure of PCs to the same dosage of S protein for 6 or 24 h did not affect either PC viability (Figure 5D) or proliferation (Figure 5E). In an *in vitro* angiogenic assay, the presence of PCs (identified by staining with a red fluorescent dye) increased the formation of CAEC networks (CAEC+PC vs CAEC monoculture, *P*<0.0001), with this response being diminished in the presence of the S protein (CAEC+PC Spike vs CAEC+PC vehicle, *P*<0.01) (Figure 5F). Strikingly, in the presence of the S protein, we observed fewer PCs localised along EC branches (Figure 5F). In contrast, the S protein did not inhibit network formation by CAECs in the absence of PCs (Figure 5F).

**Figure 6:**
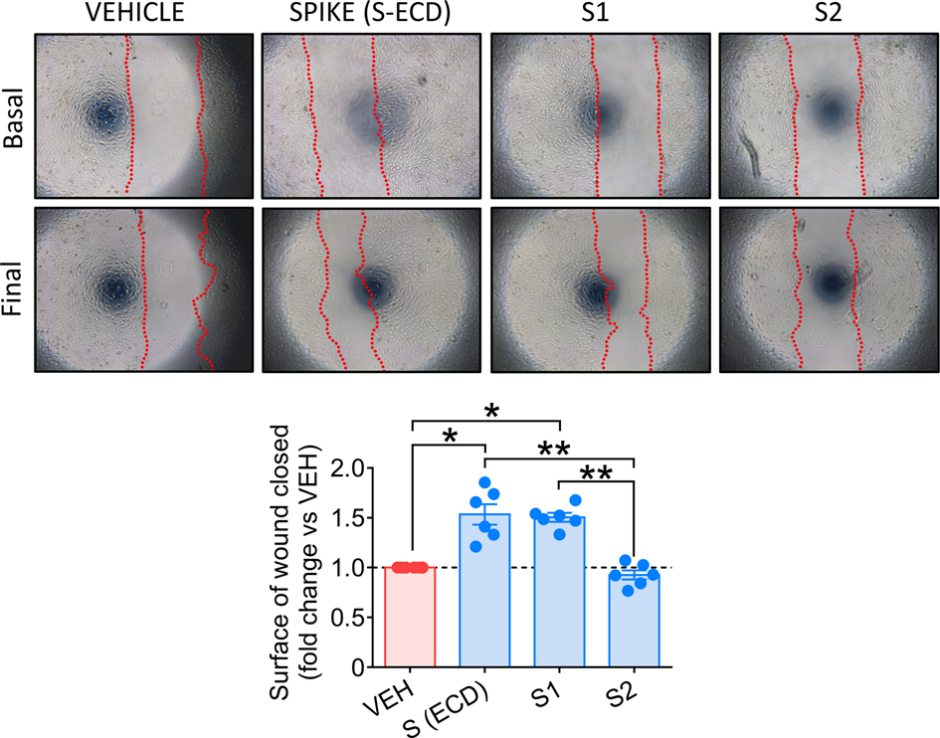
Comparison of the biological activity of the whole S-ECD and the single S1 and S2 domains in cardiac PCs, using a wound closure assay A scratch was created in confluent PCs and images taken at baseline. Cells were incubated with either S (ECD) or S1 or S2 proteins (all 5.8 nM), or vehicle for 24 h and final images were recorded. The surface of wound closure was calculated as % of the baseline area and expressed as fold-change vs vehicle. *n*=6 patients’ PCs. Graphs report individual values and means ± SEM. *P<0.05, **P<0.01.

Finally, to identify the *pathogenic* region of S protein, we compared the biological effects of the single S1 and S2 domains, along with the S (ECD) protein. As shown in Figure 6, only the S1 domain increased PC motility, reproducing the S protein effects (S and S1 vs vehicle, *P*<0.05; S and S1 vs S2, *P*<0.01).

### S protein-induced effects on cardiac PC function are CD147 dependent

**Figure 7:**
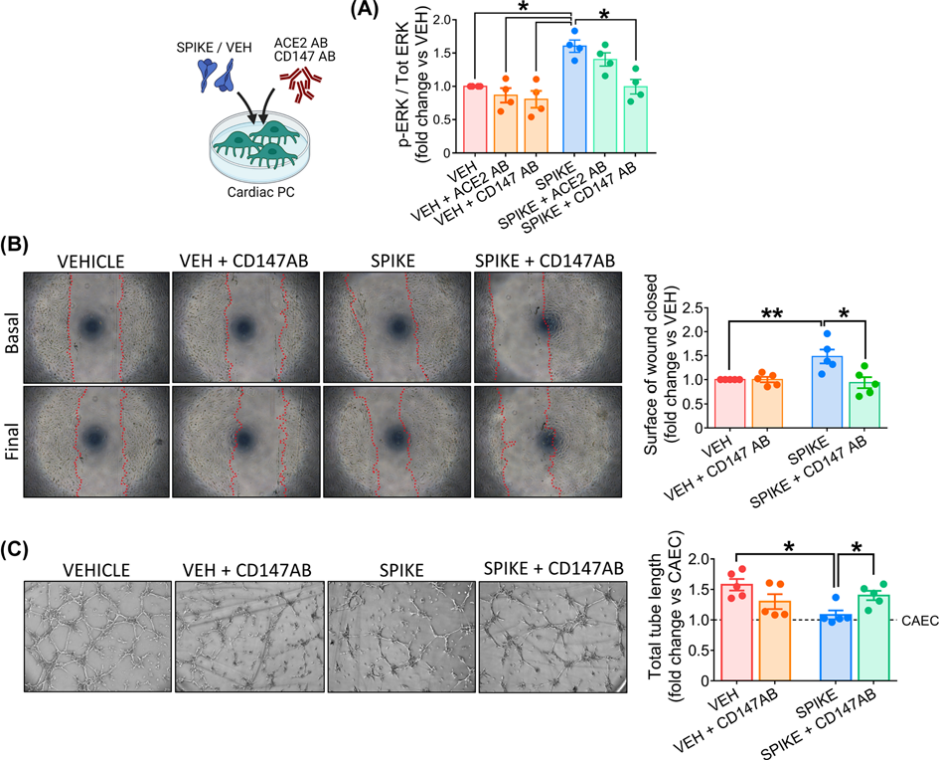
The SARS-CoV-2 S protein effects on cardiac PC function are CD147-dependent (**A**) Intracellular ERK1/2 phosphorylation/activation. Cardiac PCs were cultured for 24 h under serum and growth factors deprivation and then exposed for 1 h to the S protein (1 μg/ml – 5.8 nM) or vehicle. For receptor blockade, PCs were pre-incubated with antibodies anti-ACE2 or anti-CD147 for 1 h before the S treatment. The bar-graph reports the ratio between phospho-ERK1/2 (Thr^202^/Tyr^204^) and total ERK1/2, as measured by ELISA, and expressed as fold-change versus vehicle. *n*=4 patients’ PCs. (**B**) Migration wound closure assay. A scratch was created in confluent PCs and images taken at baseline. Where the blockade of CD147 was required, cells were pre-incubated with an antibody anti-CD147 for 1 hr. Then, the S protein (1 μg/ml – 5.8 nM) or vehicle were added to the system for 24 h. Final images were recorded. The surface of wound closure was calculated as % of the baseline area and expressed as fold-change vs vehicle. *n*=5 patients’ PCs. (**C**) Matrigel assay. Where the blockade of CD147 was required, cardiac PCs were pre-incubated with an antibody anti-CD147 for 1 h. Afterwards, CAECs and cocultures of CAECs + PCs were incubated on the top of Matrigel for 5 h, in the presence of the S protein (1 μg/ml – 5.8 nM) or vehicle. Representative images of CAECs + PCs cocultures. The bar-graphs indicate the total tube length per imaging field, expressed as fold-change vs CAEC in single culture (dotted line at *y* = 1). *n*=5 patients’ PCs. Graphs indicate individual values and means ± SEM. *P<0.05, **P<0.01.

The next step was to investigate the intracellular signalling triggered by the S protein. As shown in Figure 7A, PCs treated with the S protein had significantly increased levels of phospho-ERK1/2 (ratio P-ERK1/2 to total ERK1/2, Spike vs vehicle, *P*<0.05). A CD147 neutralising antibody (CD147AB) abolished this response (Spike vs Spike+CD147AB, *P*<0.05), while an antibody anti-ACE2 did not. As shown in Figure 7B, the CD147 blockade also prevented the S protein from inducing PC migration (Spike vs Spike+CD147AB, *P*<0.05) and inhibiting PC-CAEC network formation on Matrigel (Spike vs Spike+CD147AB, *P*<0.05, Figure 7C).

**Figure 8:**
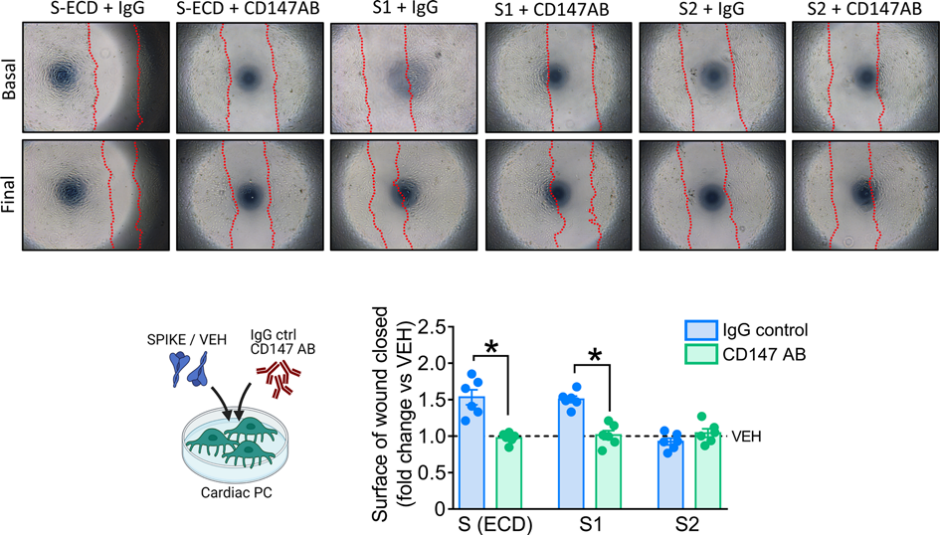
SARS-CoV-2 S and S1 proteins induction of PC motility are CD147-dependent Migration wound closure assay. A scratch was created in confluent PCs and images taken at baseline. Cells were pre-incubated with an antibody anti-CD147 or IgG isotype control for 1 h, and subsequently they were incubated with either the S (ECD), S1 or S2 proteins (all 5.8 nM) for 24 h. PBS was used as vehicle for control. Final images were recorded. The surface of wound closure was calculated as % of the baseline area and expressed as fold-change vs vehicle. *n*=6 patients’ PCs. Graphs report individual values and means ± SEM. *P<0.05.

The comparison between the S (ECD) protein and the two individual domains indicated that, similar to what was observed for the S protein, the S1 induction of PC motility is CD147-dependent (Figure 8).

**Figure 9:**
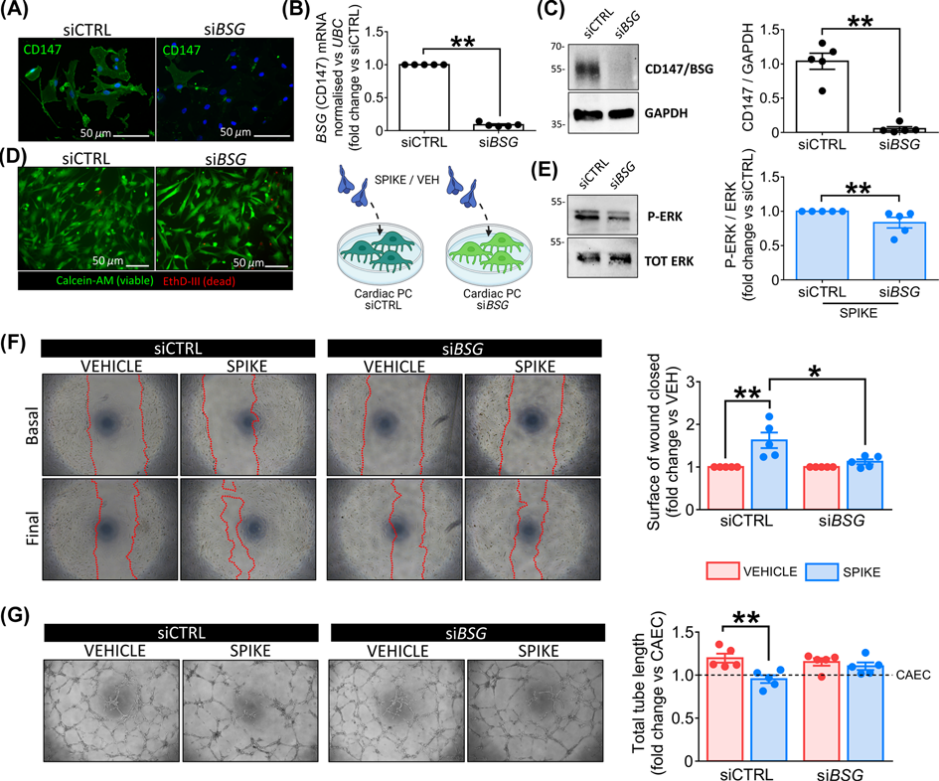
*BSG*/CD147 silencing demonstrates that the SARS-CoV-2 S protein effects on cardiac PC function are CD147-dependent (**A–C**) *BSG* silencing in PCs. CD147 protein knockdown in cardiac PCs was obtained using a pool of four small interfering RNAs (siRNAs). The silencing efficacy was confirmed using ICC (A), qPCR (B), and Western blotting (C). si*BSG*: *BSG* silencing. siCTRL: non-targeting siRNA control. (**D**) Cell viability post-silencing. In green, Calcein-AM shows the cytoplasm of live cells. The red fluorescence of EthD-III indicates the nuclei of dead cells. (**E**) Intracellular ERK1/2 phosphorylation/activation in si*BSG* PCs. Cardiac PCs were cultured for 24 h under serum and growth factors deprivation and then exposed for 1 h to the S protein (1 μg/ml – 5.8 nM) or vehicle. The bar-graph reports the ratio between phospho-ERK1/2 and total ERK1/2, as measured by Western blotting, expressed as fold-change vs siCTRL. (**F**) Migration wound closure assay. A scratch was created in confluent PCs and images taken at baseline. The S protein (1 μg/ml – 5.8 nM) or vehicle were added to the system for 24 h. Final images were recorded. The surface of wound closure was calculated as % of the baseline area and expressed as fold-change vs vehicle. (**G**) Matrigel assay. CAECs and cocultures of CAECs + PCs were incubated on the top of Matrigel for 5 h, in the presence of the S protein (1 μg/ml – 5.8 nM) or vehicle. Representative images of CAECs + PCs cocultures. The bar-graph indicates the total tube length per imaging field, expressed as fold-change vs CAEC in single culture (dotted line at *y* = 1). For all experiments, *n*=5 patients’ PCs. Graphs indicate individual values and means ± SEM. *P<0.05, **P<0.01.

To confirm the leading role of CD147 in determining the PC response to the S protein, we repeated the functional assays using cardiac PCs that were silenced for CD147/*BSG*. The silencing efficacy was confirmed using ICC (Figure 9A), qPCR (Figure 9B), and Western blotting (Figure 9C). We also verified that CD147 knockdown did not affect the cell viability (Figure 9D). When exposed to the S protein, *BSG*-silenced PCs showed less phosphorylation/activation of ERK1/2 than control cells (*P*<0.01, Figure 9E). Moreover, *BSG* silencing prevented the increase in PC motility in the presence of the S protein (si*BSG* Spike vs siCTRL Spike, *P*<0.05, Figure 9F) and rescued the pro-angiogenic activity of PCs on Matrigel (Figure 9G).

### S protein-primed PCs secrete pro-inflammatory cytokines and induce EC death

**Figure 10:**
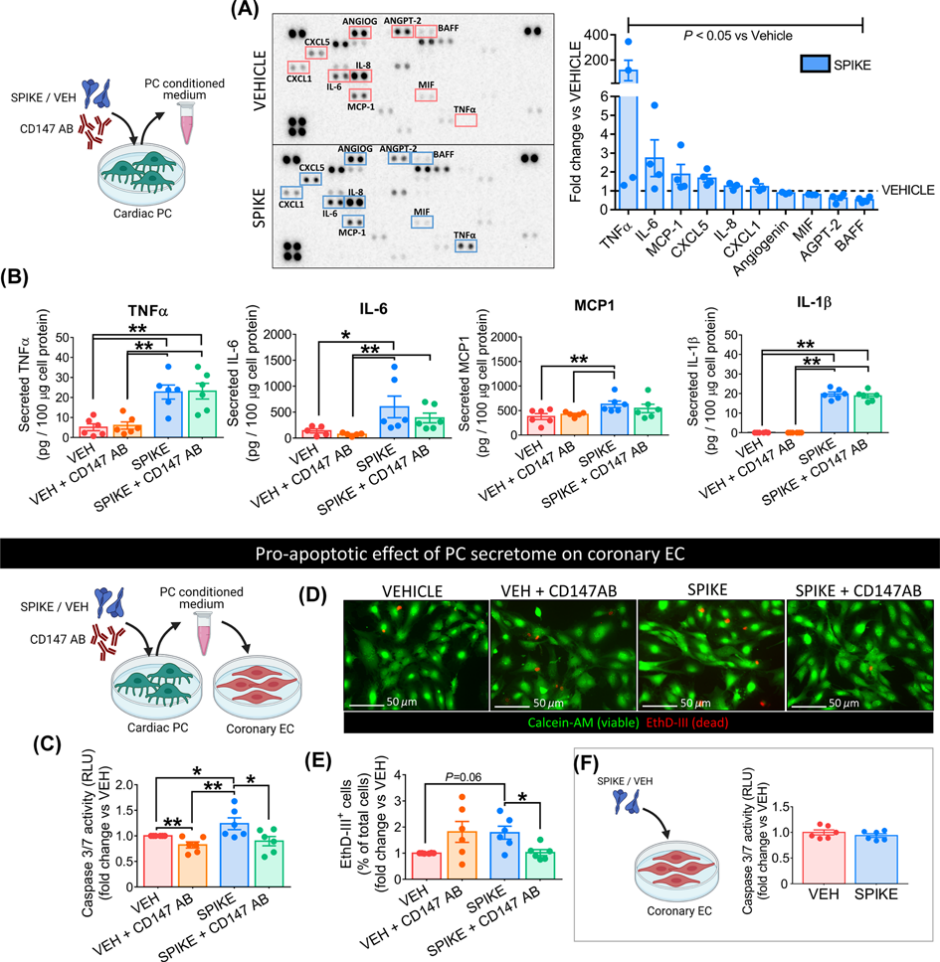
The secretome of S protein-challenged cardiac PCs is enriched in pro-inflammatory factors and induces EC death (**A**) Cytokine/chemokine protein arrays. Cardiac PCs (*n*=4 patients) were incubated with the S Protein (1 μg/ml – 5.8 nM) or vehicle for 24 h. The conditioned medium was collected and analysed with the arrays. Per each factor, average pixel densities were measured and expressed as fold-change vs vehicle (dotted line at *y* = 1). The ten factors reported in the graph were significantly regulated by Spike treatment (*P*<0.05 vs vehicle). (**B**) Quantitative ELISAs. Cardiac PCs (*n*=6 patients) were incubated with the S Protein (1 μg/ml – 5.8 nM) or vehicle for 24 h. For receptor blockade, PCs were pre-incubated with an anti-CD147 antibody for 1 h before the S treatment. The conditioned medium was collected and analysed using ELISA. Secreted factors were normalised against the total cellular proteins. (**C–E**) The pro-apoptotic effect of cardiac PC secretome on CAEC is prevented by CD147 blockade. CAECs were incubated with the PC conditioned medium for 24 h, and cell death was evaluated by measuring Caspase 3/7 activity (C, relative luminescence units (RLUs), early-stage apoptosis) and the % of cell nuclei positive for EthD-III (Calcein-AM/Ethidium Homodimer III assay – D,E – positivity for EthD-III indicates irreversible cell death). Values are expressed as fold-change vs vehicle. *n*=6 patients’ PCs. (**F**) The S protein does not cause CAEC apoptosis. Cell death in CAECs exposed to the S protein was evaluated by measuring Caspase 3/7 activity (relative luminescence units (RLUs)). All graphs show individual values and means ± SEM. *P<0.05, **P<0.01.

Finally, we assessed whether the S protein triggers cardiac PCs to release pro-inflammatory factors responsible for the *cytokine storm* [39]. The analysis of PC conditioned media using a human cytokine/chemokine protein array revealed ten factors significantly regulated by the S protein treatment (Spike vs vehicle, *P*<0.05, Figure 10A). Among these, the potent pro-inflammatory cytokines TNFα, IL-6, and MCP1 were up-regulated (average fold-change Spike vs vehicle ≥ 2, *P*<0.05, Figure 10A). We confirmed these findings using quantitative ELISAs, which also revealed that the S protein induced PCs to secrete larger amounts of IL-1β (Spike vs vehicle, *P*<0.05, Figure 10B). The CD147 neutralisation failed in preventing all these alterations (Spike vs Spike+CD147AB, not significant – Figure 10B).

Last, we checked whether the pro-inflammatory PC secretome harms EC viability. As shown in Figure 10C, exposure to the media from S protein-primed PCs induced the Caspase 3/7 activity in CAECs (Spike vs vehicle, *P*<0.05), with this pro-apoptotic effect being reduced by the anti-CD147AB (Spike vs Spike+CD147AB, *P*<0.05). These data were further confirmed by the fluorescent staining for EthD-III, indicating irreversible cell death (Figure 10D,E). Conversely, the S protein did not cause a direct pro-apoptotic effect on CAECs (Figure 10F).

## Discussion

**Figure 11:**
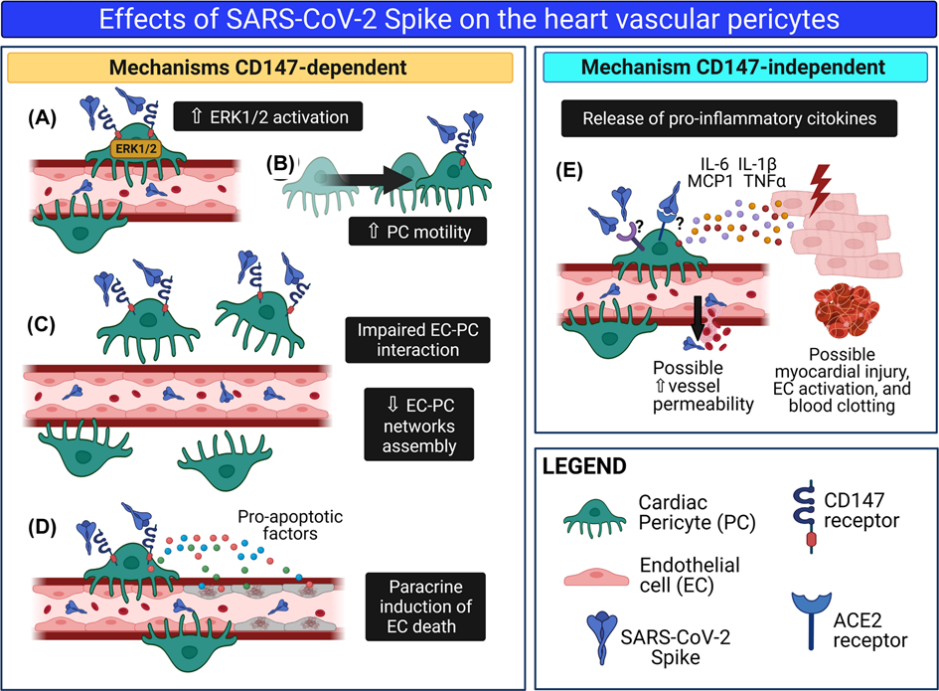
The SARS-CoV-2 S protein alters cardiac PC function Schematic summary of the research. We hypothesise that in patients with acute COVID-19, S protein molecules are cleaved from the virus particle and released from the respiratory system into the bloodstream. Through the circulation, isolated S protein reaches all organs of the body, including the heart. Here, the interaction of the S protein with the CD147 receptor on cardiac PCs triggers the ERK1/2 signalling (**A**) and provokes PC dysfunction, including increased cell motility (**B**) and decreased cooperation with coronary ECs during angiogenesis. (**C**). In addition, the S protein–CD147 interaction prompts cardiac PCs to release pro-apoptotic factors, which cause EC death (**D**). Finally, through a mechanism CD147-independent, the S protein induces PCs to release pro-inflammatory cytokines, which include MCP1, IL-6, IL-1β, and TNFα (**E**). These cytokines can damage neighbouring cardiomyocytes and activate ECs, potentially triggering blood clotting and increasing vascular permeability. This drawing was created with Biorender.com.

Our study provides novel *proof-of-concept* evidence for S protein capacity to cause molecular and functional changes in human vascular PCs, either dependently or independently of the CD147 receptor (summarised in Figure 11).

The SARS-CoV-2 virus particle consists of the three structural proteins: S, membrane (M), and envelope (E), embedded in a lipid bilayer surrounding a helical nucleocapsid comprising the viral genomic RNA bound to the N phosphoprotein. While the M and E proteins are involved in viral assembly, the S protein mediates cell entry following priming/activation by host cell proteases, primarily TMPRSS2 [20]. Moreover, the S protein activates the Raf/MEK/ERK signal transduction pathway in host cells [19]. The host ERK1/2 signalling pathway is reportedly instrumental to viral replication [19,40], and the induction of cyclooxygenase-2, a prostaglandin synthetase involved in inflammation [41]. Pharmacological inhibition or knockdown of ERK1/2 by small interfering RNAs suppressed coronavirus replication [40]. Therefore, drugs that block the binding of SARS-CoV-2 S protein to cell receptors and/or inhibit downstream signalling pathways may be potential candidates for the treatment of COVID-19.

Our study provides novel insights into the mechanism used by the virus to cause vascular damage. The classical route of infection starts with the multifunctional S protein binding to cell receptors, which opens the path to virus entry, and proceeds with manipulation of the host intracellular machinery to facilitate virus replication, assembly and egress. However, not every cell type is permissive to infection and infectivity may be variable among patients. Here, we report that two-third of patients tested did not have their PCs infected by SARS-CoV-2, while the rate of infection was below 8% in the remaining subjects, suggesting a very low permissiveness of these cells to the coronavirus, at least *in vitro*. This was confirmed using SARS-CoV-2 isolated early during the pandemic and the more transmissible SARS-CoV-2 α and δ variants (B.1.1.7 and B.1.617.2 lineages, respectively), and compared with highly infected, positive controls Caco-2-ACE2 cells. Susceptible cell lines did not differ from those that were totally resistant to virus infection regarding to the total protein levels of ACE2 and CD147 as determined using Western blot. However, we cannot exclude that subtle differences in the expression of these proteins exist at the single cell level. Further investigation in a larger population of patients is warranted to determine the cause for the inter-individual variability in PC infection. Moreover, we cannot exclude different scenarios may happen *in vivo*.

Several reports indicated that the S protein, given alone to rodents either as a soluble molecule or presented with a carrier, exerted microvascular damage and induced inflammation [6,42–44]. Moreover, a possible role for soluble S protein fragments in triggering blood clotting has been suggested [45,46]. These findings corroborate an essential biological role for the S protein beyond the presence of the whole viral particles. In addition, soluble S protein may remain engaged with cellular receptors for a longer time than the whole coronavirus, resulting in prolonged stimulation of intracellular signalling. Interestingly, our study describes an alternative non-infectious damaging mechanism triggered by the S protein alone in cardiac PCs. We challenged PCs with a concentration of S protein (5.8 nM) similar to that necessary for other natural ligands to activate ERK1/2 in PCs (EGF – 0.83 nM, and bFGF – 0.6 nM, former data from our group) [28]. Results indicate that the S protein activated PC migration and inhibited the pro-stimulatory capacity of PCs to induce CAEC networks in a Matrigel assay. The S protein did not inhibit CAEC network formation on Matrigel when these cells were challenged in the absence of PCs, thus suggesting that the S protein may activate a paracrine inhibitory mechanism affecting the PC–EC interaction. In line with this possibility, we found that the S protein induced the production of pro-inflammatory cytokines by cardiac PCs. These included MCP1, IL-6, IL-1β and TNFα, which are typical components of the *cytokine storm* associated with respiratory failure and high mortality in COVID-19 patients [39,47]. The PC pro-inflammatory secretome could potentially spread harmful effects on the surrounding vascular cells, as our experiment on CAEC apoptosis suggests. On the other hand, the S protein did not impinge upon PC viability. SARS-CoV-2 cannot replicate without the machinery of a host cell. Therefore, it could be counterproductive for the virus to kill the cell at the initial stage of the S protein engagement with host receptors.

A recent report showed that the whole S1 subunit causes the phosphorylation/activation of MEK in human pulmonary vascular cells [22]. However, using only the ACE2 RBD failed to do so. Therefore, it was not clear if the S protein signalling started from the ACE2 receptor [22]. The authors suggested that an alternative receptor, different from ACE2, might mediate the signalling of the S protein in vascular mural cells. *BSG*/CD147, a plasma membrane protein associated with oligomannosidic glycans, has emerged as a novel receptor for SARS-CoV-2 [26]. Performing several *in vitro* studies including co-immunoprecipitation assays, the authors demonstrated the direct interaction of the S protein RBD with the CD147 receptor [26]. This is in line with our findings showing that only the S1 domain, but not S2, recapitulates the CD147-dependent PC dysfunction triggered by the S-ECD. Supportive proof for the involvement of CD147 also came from research *in vivo*. In preclinical studies, transgenic expression of the human CD147 receptor conferred mice with an increased susceptibility to SARS-CoV-2 infection [48], while the administration of a neutralising antibody against the receptor successfully treated exudative pneumonia [48]. Importantly, these preclinical results were confirmed by an open-label clinical trial of meplazumab, a humanised therapeutic monoclonal antibody against CD147, which showed striking improvements in COVID-19 patients [26]. Meplazumab inhibited the interaction between CD147 and the S protein and prevented the host cells infection in a dose-dependent way [49].

Our research confirms the data from single-cell sequencing studies showing cardiac PCs express ACE2 [14,15]. However, both ACE2 and TMPRSS2 protein levels in PCs were considerably lower than those of more permissive Calu-3 and VeroE6/ACE2/TMPRSS2 cells. This difference may account for the noticeably lower virus infection of PCs. This observation is in line with recent reports showing that, *in vitro*, low ACE2 expression levels in myocardial stromal cells resulted in low susceptibility to viral infection [50], whilst mesenchymal stromal cells were not infected due to the lack of ACE2/TMPRSS2 [51]. Importantly, our data newly demonstrate that the CD147 receptor, and not ACE2, leads the S signalling in PCs. Indeed, CD147 blockade using a neutralising antibody or gene silencing, restrained the S protein from inducing ERK1/2 phosphorylation and rescued several functional features of PCs that were compromised by the S protein, including PC stability and pro-angiogenic activity. Finally, CD147 blockade protected CAECs from the paracrine apoptotic action of S protein-primed PCs. However, it failed to prevent the induction of multiple pro-inflammatory cytokines, thus suggesting the latter phenomenon involves mechanisms unrelated to the CD147 receptor in PCs.

Following replication at the site of entry, virus particles can remain localised, or can spread to other tissues, including cells resident in the heart. Establishing the kinetics of S protein *in vivo* may provide valuable diagnostic and therapeutic insights. The presence of S protein in patients’ blood was previously reported by another group [52]. In our study, low amounts of the S protein could be detected in pre-pandemic control sera. This could be explained by the sequence homology between some regions of the S protein and other human proteins/peptides. A previous report identified pathogenic regions of SARS-CoV-1 S protein, which share sequence homology with Angrgm-52 (GenBank accession number AAL62340), a novel gene up-regulated in human mesangial cells stimulated by angiotensin II and bradykinin [53]. Unfortunately, the immunogen sequence for this particular ELISA kit ab274342 is proprietary information, therefore we could not determine if it can recognise the S protein residues that have homology with unrelated peptides. To address the problem from unspecific noise, we only considered biologically relevant concentrations of S protein higher than the 95^th^ percentile of the control group. We found that 28% of the studied patients had elevated blood concentrations of S protein, most of them having been sampled at an early stage after occurrence of symptoms. The presence of S protein in the peripheral blood in the window between 5 and 10 days is compatible with subsequent neutralisation by anti-SARS-CoV-2 IgG antibodies produced by immune cells, as we documented in the same cohort of patients. High levels were more frequent in older subjects but did not associate with the severity of respiratory symptoms. However, the concentration of circulating SARS-CoV-2 and S protein may not reflect the levels present at tissue level. Moreover, risk factors and viral load may be more potent determinants of COVID-19 severity, and hence they may obscure the relative contribution of the S protein alone. Although we cannot exclude that the antibody provided in the ELISA kit also detected S protein embedded in the whole virions, two studies reported that only 12–16% of COVID-19 patients presented detectable viremia levels during the acute infection [54,55], which is half the frequency of patients we found to have high circulating levels of S protein. This comparison may suggest that shed S protein is more abundant than whole SARS-CoV-2 particles in patients’ sera.

## Study limitations

The study was conducted on isolated cells and therefore the evidence must be confirmed *in vivo*.

The amount of S protein used for *in vitro* studies was higher than the average S protein concentration detected in COVID-19 patients’ serum. However, circulating S protein represents the spill-over from infected organs, where concentration may be higher due to retention at the receptor level. Because we do not have access to post-mortem myocardial samples, we could not verify this hypothesis.

The pro-apoptotic factors responsible for EC death remain unknown and require further investigation.

## Conclusions

Although more *in vivo* investigation is needed, this work suggests that the S protein may elicit vascular cell dysfunction through CD147, independently from the infection. Blocking the CD147 receptor may help protect the vasculature of the most vulnerable patients from infection and the collateral damage caused by the S protein.

## Clinical perspectives

- COVID-19 manifests as a microvascular syndrome, but whether SARS-CoV-2 infects and damages heart vascular PCs remains unknown.
- We provide evidence that cardiac PCs are rarely infected by SARS-CoV-2. Importantly, we show that a recombinant S protein alone elicits cellular signalling through the CD147 receptor in cardiac PCs, thereby inducing cell dysfunction and microvascular disruption *in vitro*. We also document that circulating levels of S protein were elevated in some patients with COVID-19.
- The present study suggests that soluble S protein can potentially propagate damage to organs distant from sites of infection, promoting microvascular injury. Blocking the CD147 receptor in patients may help protect the vasculature not only from infection, but also from the collateral damage caused by the S protein.

## Data Availability

The data underlying this article will be shared on reasonable request to the corresponding authors.

## Abbreviations

ACE2: angiotensin-converting enzyme 2
*BSG*: Basigin
CAEC: coronary artery endothelial cell
COVID-19: coronavirus disease-2019
DAPI: 4′,6-diamidino-2-phenylindole
dsRNA: double-stranded RNA
EC: endothelial cell
ECD: extracellular domain
ERK1/2: extracellular signal-regulated kinase 1/2
EthD-III: Ethidium Homodimer III
HIS: hexahistidine
ICC: immunocytochemistry
MCP1: monocyte chemoattractant protein-1
MOI: multiplicity of infection
NG2: Neural/Glial antigen 2
PBS: phosphate-buffered saline
PC: pericyte
PDGFRβ: platelet derived growth factor receptor β
PFA: paraformaldehyde
RBD: receptor-binding domain
S: spike protein
SARS-CoV-2: severe acute respiratory syndrome coronavirus 2
SEM: standard error of the mean
TMPRSS2: transmembrane serine protease 2
TNFα: tumour necrosis factor
