# Correction: The SARS-CoV-2 Spike protein disrupts human cardiac pericytes function through CD147 receptor-mediated signalling: a potential non-infective mechanism of COVID-19 microvascular disease

**DOI:** 10.1042/CS-2021-0735_COR

**Published:** 2024-04-04

**Keywords:** angiotensin converting enzyme 2, CD147, COVID-19, Microvascular disease, pericyte, Spike protein

The authors of the original article “The SARS-CoV-2 Spike protein disrupts human cardiac pericytes function through CD147 receptor-mediated signalling: a potential non-infective mechanism of COVID-19 microvascular disease” (DOI: 10.1042/CS20210735) would like to correct Figure 8 of their paper.

**Figure 8:**
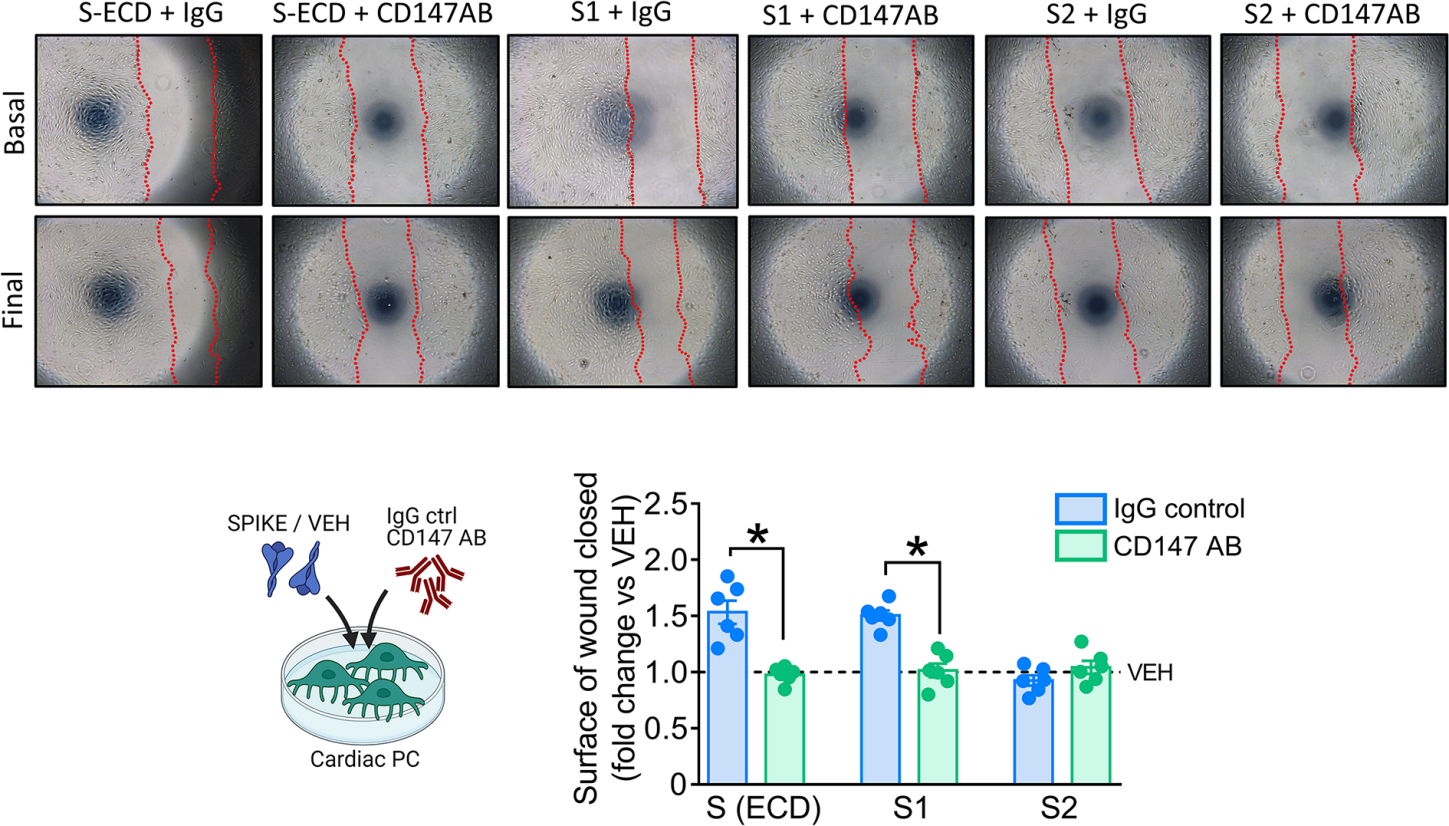
SARS-CoV-2 S and S1 proteins induction of PC motility are CD147-dependent

The authors state that two images from the SPIKE (S-ECD) group in Figure 6 were inadvertently included in the S1 + IgG panel in Figure 8 resulting in an unwanted duplication. The authors apologise for this error and state this does not impair the correctness or validity of bar and dot graphs and the related statistical analysis presented in Figure 8.

The requested correction has been assessed and agreed by the *Clinical Science* Editorial Board and Editor-in-Chief. The authors declare that these corrections do not change the conclusions of their paper. The corrected version of Figure 8 is presented here:

